# Cervico-Dorsal Intramedullary Spinal Cord Abscess with *Aspergillus fumigates* following Pulmonary Infection in an Immunocompetent Patient

**DOI:** 10.3390/medicina59040806

**Published:** 2023-04-20

**Authors:** Vasile Deniss Mereuta, Anca Sava, Cristinel Ionel Stan, Lucian Eva, Gabriela Florenta Dumitrescu, Nicolaie Dobrin, Cornelia Tudorache, Alexandru Chiriac, Irina Ruxandra Strambu, Dragos Andrei Chiran, Ana Maria Dumitrescu

**Affiliations:** 1 Department of Oral and Maxillofacial Surgery, St. Spiridon” Emergency Clinical County Hospital, 700111 Iași, Romania; 2 Department of Morpho-Functional Sciences I, “Grigore T. Popa” University of Medicine and Pharmacy, 700115 Iași, Romania; 3 Department of Pathology, “Prof. Dr. N. Oblu” Emergency Clinical Hospital, 700309 Iași, Romania; 4 2nd Neurosurgical Clinic, “Prof. Dr. N. Oblu” Emergency Clinical Hospital, 700309 Iași, Romania; 5 Department of Radiology, “Prof. Dr. N. Oblu” Emergency Clinical Hospital, 700309 Iași, Romania; 6 “Marius Nasta” Institute of Pneumology, 050159 Bucharest, Romania; 7 Department of Pneumology, “Carol Davila”, University of Medicine and Pharmacy, 020021 Bucharest, Romania

**Keywords:** *Aspergillus fumigates*, spinal cord abscess, community pneumonia, antifungal agent

## Abstract

Invasive forms of aspergillosis of the nervous system are relatively rare and are usually diagnosed in immunocompromised patients. We present the case of a young female patient, treated in the last two months with corticosteroids and antifungal drug for pulmonary aspergillosis, who developed progressive paraparesis. An intramedullary abscess at the C7–D1 level was identified and the lesion was treated with a combination of surgery and antifungal therapy. Histopathologic findings of surgical specimens showed myelomalacia with *Aspergillus* hyphae and a peripheral rim of neutrophils. We consider that the use of multiple drugs and corticosteroids for our patient’s initial community pneumonia could be the factor that transformed her into a mildly immunocompromised individual and permitted the *Aspergillus* spp. to disseminate through the blood and into the spinal cord. Moreover, we highlight the fact that more attention should be paid to living and working conditions of the patients, as a simple colonization of the lung with *Aspergillus* spp. could develop, in a short time, into an invasive disease with a high risk of mortality.

## 1. Introduction

Aspergillosis represents an infection given by fungi of *Aspergillus* species (spp.), which have no specific geographic distribution. The genus *Aspergillus* comprises of more than 300 species, but only a few of them cause human infections, mostly following environmental exposure [1]. *Aspergillus* spp. live in the soil, but abundantly sporulate, with formation and release of numerous conidia into the atmosphere. Their main entrance in the human body is the sino-respiratory tract. These conidia have a small diameter (2–3 microns), and by inhalation they reach the pulmonary alveoli, after which they can spread through the blood to other locations, such as skin [2], peritoneum [3], kidneys, bones, eyes, heart, gastrointestinal tract, liver, spleen, pancreas, and thyroid [4,5], but dissemination in the central nervous system appears to be in second place after respiratory infection.

Inhalation of conidia by immunocompetent individuals rarely causes illness because conidia are eliminated by innate immune mechanisms. However, since the number of immunocompromised patients has increased in recent years, an increasing number of cases with *Aspergillus* infection are being reported.

The spectrum of pulmonary aspergillosis depends upon the host immune status because there are reports about non-invasive and invasive forms. Immunocompetent individuals can present simple colonization or allergic bronchopulmonary aspergillosis. Immunocompromised patients can be affected by chronic pulmonary aspergillosis (represented by simple aspergilloma, chronic cavitary pulmonary aspergillosis, chronic fibrosing pulmonary aspergillosis, and *Aspergillus* nodule), locally invasive aspergillosis (subacute invasive aspergillosis, airway invasive aspergillosis), or angioinvasive aspergillosis with hematogenous dissemination in other organs or systems [6,7].

Invasive aspergillosis with extrapulmonary involvement is a rare condition. Its most common clinical manifestations are invasive sinusitis, fungaemia, multiple brain abscesses, osteomyelitis, or epidural abscess [8]. Invasive aspergillosis usually occurs in immunocompromised hosts, such as those with hematological malignancies, hematopoietic stem cell or solid organ transplants, congenital or acquired immunodeficiency, collagen vascular disease, sarcoidosis, as well as use of corticosteroids and other immunosuppressive drugs [9].

We present the case of a young immunocompetent female patient who developed an invasive pulmonary and spinal cord aspergillosis with poor evolution despite treatment with a combination of surgery and antifungal therapy. We described the clinical, radiological, and laboratory features of the disease and compared them with data from the literature, which is reviewed.

## 2. Detailed Case Description

A 34-year-old Caucasian female was admitted in our hospital in January 2019 with progressive and severe neurological deficits of the lower limbs that appeared in the last week.

During neurological examination, the patient was conscious, cooperative, and presented hyperesthesia on her left hemithorax, and severe motor deficits (3/5 bilateral brachial muscles strength and 2/5 bilateral crural muscles strength on the Medical Research Council Manual Muscle Testing Scale), with spastic character (Grade 3 on the Modified Ashworth Scale). The walking test could not be realized, the Lasègue test was negative, and the neurological examination revealed the following: absent abdominal cutaneous reflexes, hyperspastic osteo-tendinous reflexes, and bilateral positive Babinski’s sign. The patient had urinary retention, requiring transurethral catheterization (à demeure).

She was a former smoker, who lived for several months in an environment with humidity and mold while working in another country.

**Figure 1:**
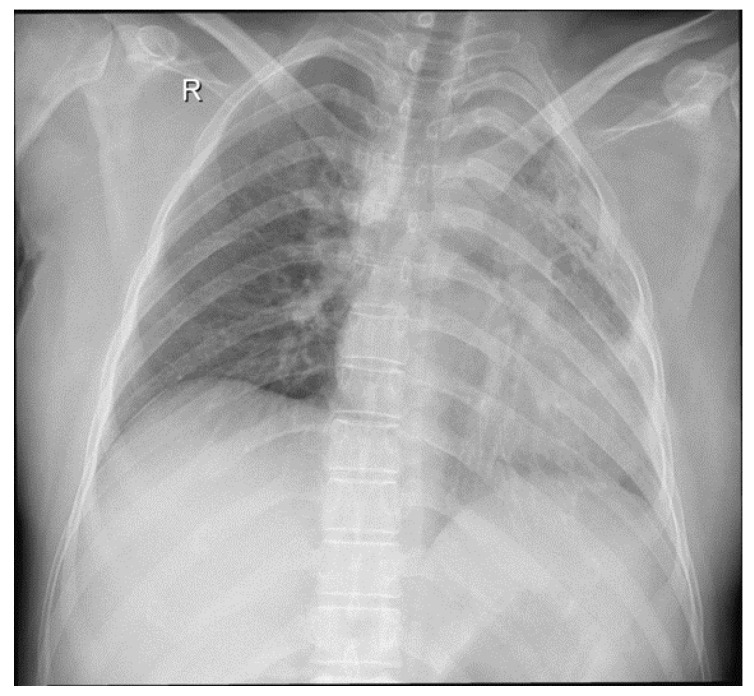
Chest radiograph: left pulmonary opacities, fibrosis, and bronchiectasis. Dense consolidation in the left upper lobe with clear evidence of volume loss.

**Figure 2:**
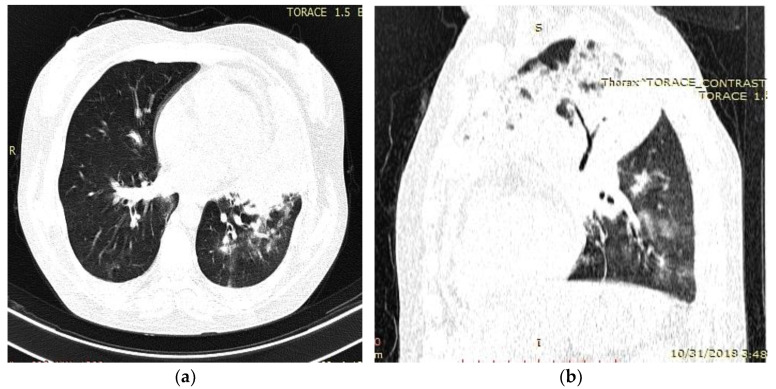
Chest computed tomography (CT) findings (October 2018): axial (**a**) and sagittal (**b**) lung window settings showed massive inhomogeneous consolidation in the entire left upper lobe, with air bronchogram identification. Mild pericardial effusion, and few alveolar opacities in left, lower, and middle lobes.

Her medical history revealed an admission in a county hospital in October 2018 for general malaise, malnutrition, pale skin, fever, left thoracic pain, cough, difficulty in expectoration, then hemoptysis, and dyspnea. Chest radiographs (Figure 1) revealed a relatively homogeneous opacity in the upper left pulmonary lobe. Chest Computer Tomography (CT) showed alveolar consolidation of the left upper pulmonary lobe, minimal bilateral pleural effusion, a nodule in the upper right pulmonary lobe, and mildly pericardial fluid accumulation (Figure 2a,b).

Laboratory analysis identified important anemia (erythrocytes = 2.7 × 10^6^/mm^3^, Hemoglobin = 8.1%, hematocrit = 28.9%), severe thrombocytosis (788 × 10^3^/μL), and severe inflammatory syndrome (leukocytes = 30.5 × 10^3^/μL, neutrophils = 26.35 × 10^3^/μL, fibrinogen = 791 mg/mL, Erythrocytes Sedimentation Rate (ESR) = 115 mm/h) (Table 1). C-reactive protein was not performed since it is considered a second-line inflammation marker, as per local hospital protocol. The first-line markers were extremely high (ESR), with high fibrinogen and leukocytes count values to identify the inflammatory syndrome. Antibodies to HIV-1 and HIV-2 were not detected by enzyme-linked immunosorbent assay (ELISA).

Sputum smear microscopy showed rare epithelial cells, rare erythrocytes, numerous neutrophils, and Gram-positive diplococci. Mycological analyses of sputum specimen revealed some yeasts, but fungal culture was negative. Sputum microscopy using Ziehl–Neelsen stain did not find any acid-fast bacilli.

A suspicion of superior left lobar community-acquired pneumonia was raised, and the patient was initially treated with oral meropenem (3 g/day), then oral levofloxacin (1 g/day) accompanied by amikacin (500 mg/day), and corticotherapy with dexamethasone.

Clinical, biological, and radiological course was unfavorable with intense dyspnea, small hemoptysis, bilateral pleural effusion, and acute respiratory failure.

After two weeks, in November 2018, the patient was transferred to a thoracic surgery unit for further investigation and treatment. A new bacterioscopy of sputum was performed. It revealed Gram-positive diplococci and one *Aspergillus* spp. hypha. Sputum culture for acid-fast bacilli (AFB) and pyogenic organism were negative.

A bronchoscopic examination with bronchial aspiration was performed. No particular morphological change was seen in the endobronchial epithelium. The laboratory analysis of the bronchial aspirate revealed the following:

1. Gram-stained smears showed an intense inflammatory reaction with neutrophils;
2. Ziehl-Neelsen-stained smear was negative for acid fast bacilli;
3. Culture for *Mycobacterium tuberculosis* was negative;
4. Twenty colonies of *Aspergillus* spp. were identified in fungal culture.

According to clinical, radiological, and microbiological findings, a diagnosis of a left superior lobar pneumonia with *Aspergillus* spp. was established and the patient was transferred to the “Marius Nasta” Institute of Pneumology, Bucharest, Romania.

A new bronchoscopy revealed the suppurative aspect of the left upper lobe, and the bronchial aspirate revealed rare bronchial epithelial cells, numerous neutrophils, rare diplococci, and rare mycelium filaments. Fungal culture showed colonies of filamentous fungi identified as *Aspergillus fumigates*.

A leukemoid reaction with neutrophilia (leukocytes = 29 × 10^3^/μL, neutrophils = 28.27 × 10^3^/uL), severe thrombocytosis (817 × 10^3^/μL), and an inflammatory syndrome (Erythrocytes Sedimentation Rate (ESR) = 115 mm/h) were identified (Table 1), but bone marrow smear was devoid of any blasts. The patient was treated with two antibiotics (cefixime and doxycycline, for 14 days) and one antifungal agent (itraconazole, for the next two months). Clinical evolution was favorable, but mild leukocytosis with neutrophilia and modest inflammatory syndrome (fibrinogen = 399 mg/dL, ESR = 75 mm/h) still persisted.

After two months of antifungal treatment, the patient had good recovery from a pulmonary point-of-view, but suddenly, during the first days of February 2019, she became unable to walk without a support. As such, she was admitted in our Neurosurgery Department.

**Figure 3:**
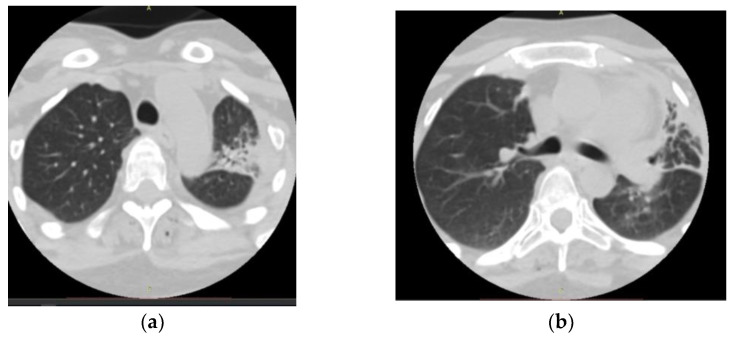
Chest computed tomography (CT) findings (February 2019): axial lung window setting showed atelectasis and a solid nodule surrounded by a halo of ground-glass attenuation in the left lung (superior segment and lingula), representing the “CT halo sign”. (**a**), axial section through trachea; (**b**), axial section below carina level.

On admission, a chest computed tomography (CT) revealed atelectasis and a solid nodule surrounded by a halo of ground-glass attenuation in the left lung, representing the “CT halo sign” (Figure 3a,b).

**Figure 4:**
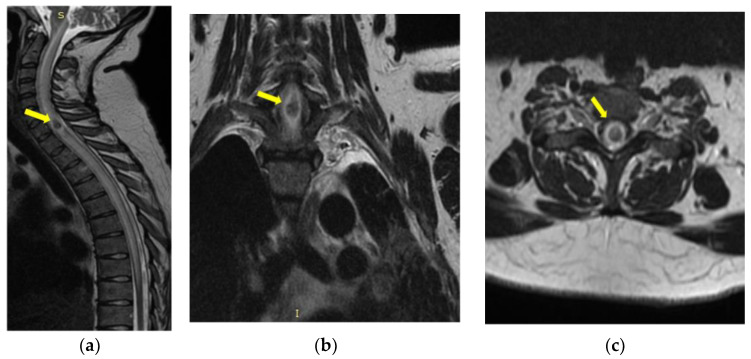
MRI of the cervical spine T2-weighted image—sagittal (**a**), coronal (**b**), and axial (**c**) sections: cord expansion with a decreased signal from abscess core and increased signal from surrounding edema. The yellow arrow indicates the expansive cystic intramedullary process.

**Figure 5:**
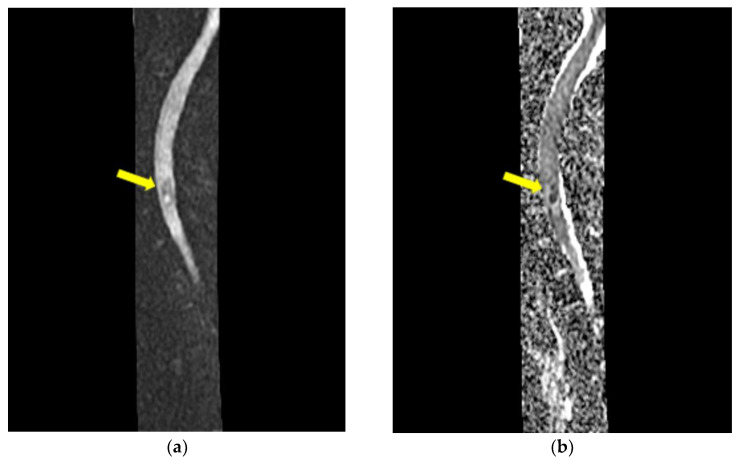
MRI of the cervical spine—sagittal DWI (**a**) and ADC map (**b**): the lesion is hyperintense on DWI, and restricted diffusion is illustrated on ADC map. The yellow arrow indicates the expansive cystic intramedullary process.

**Figure 6:**
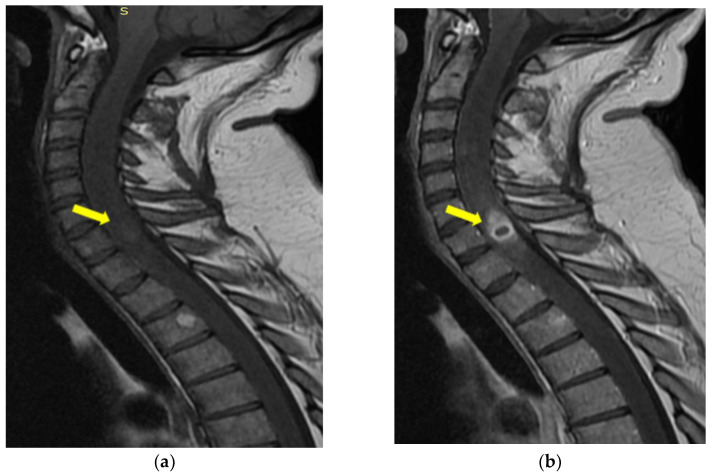
MRI of the cervical sagittal spine T1-weighted image (**a**) and after intravenous Gd-chelates (**b**): ring-enhancing mass within cord. The yellow arrow indicates the expansive cystic intramedullary process.

Magnetic resonance imaging (MRI) of the cervico-dorsal spinal cord showed diffuse swelling of the cord, with a hypointense area with marginal enhancement within the cord at the C7–T1 (Figure 4, Figure 5 and Figure 6). The final imaging diagnosis was of an expansive cystic intramedullary process (tumor or abscess), located at the C7–D1 level, with peripheral contrast uptake and moderate perilesional edema with a diameter of approximately 1.2 cm.

Routine blood investigations revealed mild leukocytosis and low inflammatory syndrome (ESR = 65 mm/h) (Table 1). Cerebro-spinal fluid showed normal appearance and only 5 cells/mm3 upon microscopic examination.

In agreement with the patient and the family, a surgical exploration was proposed for decompressive and diagnostic purposes. The patient underwent surgical decompressive laminectomy, but, due to the vascular and electrophysiological monitoring aspects, a biopsy was first performed. Later, a ”second look” was realized on the following day, with a subtotal resection of the lesion at the C7 level. The microscopical intraoperative aspect was of an intramedullary lesion with a dense gray border and a softening of the central tissue. This lesion presented strong adhesion to the adjacent medullary tissue.

**Figure 7:**
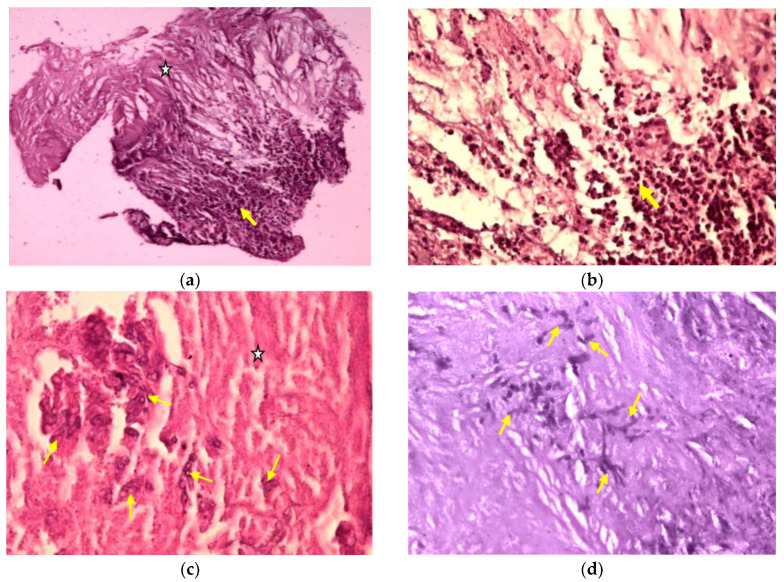
Microphotographs: (**a**) Spinal cord histological architecture was replaced by an extensive area of coagulative necrosis (white star), surrounded by dense acute inflammatory infiltrate (yellow arrow) (HE, ×100). (**b**) The same histological area with a higher magnification showed the necrotic area being surrounded mainly by numerous neutrophils (yellow arrow) (HE, ×400). (**c**) Another specimen showed extensive coagulative necrosis with clusters of hyphae inside (HE, ×400). (**d**) The same histological area, stained with Periodic acid–Schiff (PAS) staining method, showed colonies of dichotomous angle branching hyphae of *Aspergillus* spp. embedded in the necrotic material (PAS, ×200).

Both specimens were sent to the Department of Pathology of the same hospital, where they were processed using standard histological technique (fixation in 4% neutral buffer formalin, paraffin embedding, cutting the paraffin blocks at 3μm). Histological sections were stained with hematoxylin-eosin and Periodic acid-Schiff (PAS). Histopathological examination of the first sample identified spinal cord tissue with edema and numerous corpora amylacea. The second sample revealed a large area of coagulative necrosis surrounded by numerous inflammatory cells (Figure 7a), with a predominance of neutrophils (Figure 7b). In the area of necrosis, we identified colonies of dichotomous branching at acute angles of the hyphae (consistent with *Aspergillus* spp.) (Figure 7c), which showed positivity for PAS staining (Figure 7d). The final pathological diagnosis was a spinal cord abscess with *Aspergillus* spp.

No improvement in the patient’s neurological status was observed after surgery. A physical therapy rehabilitation program was initiated, and the patient has been discharged with the recommendation to follow the treatment with antifungal agent (Itraconazole) for the next two months. Our patient did not return to the clinical and radiological control for the next three years, which is why we assume that she may have died.

## 3. Discussion

Spinal infection with *Aspergillus* spp. is uncommon, and spinal cord abscess is even rarer, but represents a major cause of mortality.

Spinal aspergillosis can develop by contiguity, hematogenous spread, or direct implantation of the fungus, e.g., traumatic or iatrogenic [10].

Primary aspergillosis of the spine occurs due to an external source. This is the case for spinal surgical procedures in an operating room that have *Aspergillus* conidia in their atmosphere [11,12], or if spinal maneuvers are performed using instruments, plastic syringes, needles, cannulas, and ampules of anesthetic agents that are contaminated with *Aspergillus* spp. This situation can be possible if these supplies had been stored in a humid warehouse with high concentrations of *Aspergillus* conidia in the environment, such as what happened after the catastrophic floods in Sri Lanka (2004) or New Orleans (2005) [13,14].

Hospitalized patients can also develop invasive aspergillosis due to insanitary conditions of the care practice, hospital renovation work in the vicinity of immunocompromised patients, overuse or misuse of steroids and broad-spectrum antibiotics, or use of contaminated infusion sets/fluid [15].

The species of *Aspergillus* encountered in spinal infection are *Aspergillus flavus* [11] and *Aspergillus nidulans* [16], but *Aspergillus fumigates* is the most common species found in this location [17].

Invasive aspergillosis of the central nervous system is developed most commonly in immunocompromised individuals, i.e., previous bone marrow transplantation, prolonged neutropenia (e.g., following cytotoxic regimens for acute leukemia [15], or that appearing in advanced AIDS), immunosuppression (steroid use) for hematopoietic stem cell transplant and solid organ transplant recipients, thermal burns, or hepatic failure [18]. This disease can also occur in mildly immunocompromised individuals, presenting chronic alcoholism, malnutrition, or comorbidities, including diabetes, chronic liver disease, connective tissue diseases, such as rheumatoid arthritis and ankylosing spondylitis, bronchiectasis, or prior cavitary tuberculosis [19,20,21,22].

However, in very rare cases, invasive aspergillosis of the central nervous system can develop in immunocompetent individuals [10]. Maybe, in these cases, the environmental factors play a key role in the pathogenesis of this infection [23].

Our patient did not have any immunocompromised conditions. Additionally, her HIV status was negative. Reviewing her past medical history, we can hypothesize that our patient probably developed community-acquired pneumonia of a pulmonary lobe previously colonized by *Aspergillus fumigates*, as she previously worked, for several months, in humid spaces doing renovation activities of an old hotel, in a country outside of her residence. Probably, there was an environment with exposure to large quantities of *Aspergillus* spp. Additionally, for her initial community-acquired pneumonia, she was treated with a large spectrum of antibiotics and corticosteroids, and this treatment could be the cause for the subsequent pulmonary and spinal cord abscesses due to *Aspergillus fumigates*. However, the spinal cord abscess was the consequence of hematogenous dissemination of the fungi, initially located in her lungs.

In *Aspergillus* pneumonia, chest radiography shows only nonspecific changes in the early stages of disease, and pleural effusions are uncommon [24]. However, our patient presented mildly bilateral pleural effusion and pericarditis.

Prolonged neutropenia is generally thought to be the major factor for invasive pulmonary aspergillosis [25], but there are also a few articles about a leukemoid reaction in aspergillosis, and this condition was considered to be a reaction to fungal infection [26]. Our case presented a leukemoid reaction with neutrophilia and severe thrombocytosis, which can be consider markers of an abnormal inflammatory reaction to an association between a bacterial and fungal infection.

On the other hand, the most common CT abnormalities in lung aspergillosis are cavity and nodules, but consolidation appears in very few cases [27]. Our case presented the least common aspect, i.e., the alveolar consolidation with abscess formation.

Definitive diagnosis of a pulmonary aspergillosis depends on the demonstration of casual agents in sputum smear microscopy and culture. In our case, the identification of one hypha of *Aspergillus* spp. raised the suspicion of a fungal infection, but the development of filamentous colonies in culture established the diagnosis. Additionally, bronchial aspirate is proven to be a good method for hyphae identification.

The literature mentions several cases of spinal aspergillosis, but most of them were vertebral osteomyelitis and spinal epidural abscesses, especially in immunocompromised patients, due to HIV infection [28].

Until now, there are few reports of a spinal cord infection with *Aspergillus* spp. [18,21,29,30].

In 2007, Karakousis et al., reported the case of a 40-year-old woman with a history of multiple sclerosis, asthma, and Crohn disease, who suddenly developed paraparesis. MRI revealed an edematous spinal cord, from C5 through the conus, and multiple enhancing extramedullary masses. Spinal cord biopsy revealed a caseating granuloma with fungal hyphae, morphologically consistent with *Aspergillus* species, but fungal cultures were negative. The patient was treated for 7 months with voriconazole and caspofungin, and then with posaconazole for another nine months, until she remained clinically stable [29].

McCaslin et al. also reported the case of a 19-year-old woman with active acute lymphoblastic leukemia, who presented fever, neurologic deficits, and intramedullary *Aspergillus* abscess at T12–L1, resulting from adjacent vertebral osteomyelitis [21].

These two cases of spinal cord aspergillosis developed in young female patients. Our patient was also a young woman with intramedullary *Aspergillus* abscess, and as such it added knowledge about this disease.

There are also some other reports of spinal cord aspergillosis. Sheth et al. reported the contiguous extension of *Aspergillus* infection from a pulmonary aspergilloma to the epidural and subdural spaces and spinal cord. Histopathologic findings of the spinal cord showed *Aspergillus* hyphae penetrating the myelin sheath and myelomalacia, predominantly in the anterior and lateral columns [30]. On the other hand, Nakazato et al. reported massive spinal cord necrosis caused by *Aspergillus* spp. in a patient with adult T-cell leukemia and pulmonary aspergilloma [18].

The diagnosis of a spinal cord aspergillosis is more difficult because CSF often proved to be normal [31]. In this situation, the diagnosis depends on the demonstration of casual agents in tissue samples [11]. From a morphological point of view, *Aspergillus* spp. has septate hyphae, which show dichotomous branching that has the tendency for angioinvasiveness. Hyphal elements block spinal cord blood vessels, leading to infarct, commonly hemorrhagic. This sterile infarct becomes septic when *Aspergillus* spp. penetrate the vessel wall into the ischemic nervous tissue [32].

We can consider that our patient fulfilled the diagnostic requirements for invasive pulmonary and spinal cord aspergillosis in an immunocompetent individual, as she presented the following characteristics:

1. Three-month duration of pulmonary symptoms;
2. Radiological evidence of chronic pulmonary lesion;
3. Mycological demonstration of *Aspergillus fumigates* hyphae by microscopy from spu-tum, microscopy and culture from bronchial aspirate, and histopathological evi-dence of the presence of *Aspergillus fumigates* hyphae with dichotomous branching in the spinal cord biopsy;
4. Exclusion of other pulmonary pathogens, such as tuberculosis mycobacteria;
5. Lack of major discernible immunodeficiency, such as AIDS, leukemia, or organ trans-plant, but working in a humid space with molds on its walls.

Regarding the treatment of spinal cord aspergillosis, there is little experience in the field. Vertebral osteomyelitis and epidural abscess due to *Aspergillus* spp. should be treated with aggressive surgical debridement and aggressive antifungal therapy [16]. As fungal agents, voriconazole can be used with intravenous administration for 6 months, but 12-week mortality can be high [32,33].

However, spinal cord aspergillosis does not yet have a well-established treatment. Perhaps the model of treatment of the cerebral abscess with *Aspergillus* spp. [34] can also be applied in the treatment of spinal cord abscess with voriconazole over the following two years from the surgery time.

## 4. Conclusions

Aspergillosis is quite rare among Central Nervous System infections, with the majority of cases having a fatal prognosis due to immunosuppression and the reduced penetrability of antifungal drugs through the blood–brain barrier.

Our case presentation added information on invasive aspergillosis in immunocompetent patients. However, the use of multiple drugs and corticosteroids to treat our patient’s initial community pneumonia could be the factor that transformed her into a mildly immunocompromised patient, and permitted the *Aspergillus fumigates* to disseminate through the blood and into the spinal cord. Moreover, we highlight the fact that more attention should be paid to living and working conditions of the patients, as a simple colonization of the lung with *Aspergillus* spp. could quickly develop into an invasive disease with a high risk of mortality, even in immunocompetent individuals.

## Figures and Tables

**Table 1:** Evolution of the biological parameters during the evolution of the disease.

Laboratory Analysis	1st Admission (County Hospital, Focsani)	2nd Admission (Thoracic Surgery Unit, Iasi)	3rd Admission (Institute of Pneumology, Bucharest)	4th Admission (Neurosurgery, Iasi)
Erythrocytes	2.7 × 10^6^/mm^3^	2.6 × 10^6^/mm^3^	1.86 × 10^6^/mm^3^	3.89 × 10^6^/mm^3^
Hemoglobin	8.1 g/dL	6.5 g/dL	5.3 g/dL	12 g/dL
Hematocrit	28.9%	24%	17.5%	35.6%
Leucocytes	30.5 × 10^3^/mm^3^	35.67 × 10^3^/mm^3^	29 × 10^3^/mm^3^	11.23 × 10^3^/mm^3^
Neutrophils	86.4% (20.35 × 10^3^/μL)	85.5% (30.49 × 10^3^/μL)	95.7% (28.27 × 10^3^/μL)	62.9% (7.06 × 10^3^/μL)
Basophiles	2.3%	0%	0.1%	0.6%
Eosinophils	0.5%	0.1%	0.1%	1.8%
Monocytes	4%	4.1%	1.8%	4.3%
Lymphocytes	5.7%	10.3%	2.3%	26.4%
Thrombocytes	788 × 10^3^/μL	1130 × 10^3^/μL	817 × 10^3^/μL	388 × 10^3^/μL
Fibrinogen	791 mg/mL	not available	not available	399 mg/dL
ESR	115 mm/h	140 mm/h	160 mm/h	68 mm/h
Antibodies to HIV-1 and HIV-2	negative	not available	negative	negative

## Data Availability

Not applicable.
